# All-Solid-State Flexible Asymmetric Supercapacitor with Good Cycling Performance and Ultra-Power Density by Electrode Materials of Core-Shell CoNiO_2_@NiAl-Layered Double Hydroxide and Hollow Spherical α-Fe_2_O_3_

**DOI:** 10.1186/s11671-019-2910-5

**Published:** 2019-03-11

**Authors:** Jijun Zhang, Zexiang Chen, Yan Wang, Xinyu Yan, Zhiyu Zhou, Huifang Lv

**Affiliations:** School of Optoelectronic Science And Engineering, University of Electronic Science and Technology of China, North Jianshe Road 4, Chengdu, 610054 China

**Keywords:** Asymmetric supercapacitors, Rate capability, CoNiO_2_, NiAl-LDH, α-Fe_2_O_3_, State electrolyte

## Abstract

High electrochemical performance of asymmetric supercapacitor (ASC) depends on exquisite nanostructure design and synthesis of electrodes, including structural controllable design and synthesis of high theoretical performance materials and nanocomposite materials. Herein, a template-free hierarchical core-shell nanostructure of CoNiO_2_@NiAl-layered double hydroxide (NiAl-LDH) and α-Fe_2_O_3_ with a hollow spherical structure composed of nanoparticles are successfully prepared. The CoNiO_2_@NiAl-LDH as the cathode electrode and the hollow spherical α-Fe_2_O_3_ as the anode electrode of the ASC devices exhibit superior electrochemical performance. The gel of polyvinyl alcohol (PVA) and KOH acts as the solid electrolyte and the separator to assemble into the all-solid-state flexible ASC devices. Two of the CoNiO_2_@NiAl-LDH//α-Fe_2_O_3_ ASC devices in series are fabricated to meet the voltage requirement of mobile energy equipment, which exhibit a maximum energy density of 65.68 Wh kg^−1^ at the power density of 369.45 W kg^−1^. Interestingly, in addition to many advantages that the solid electrolyte in ASC devices already have, we find that it can be an alternative way of solving the problem of iron oxide cycling performance, and of course it can also be used as a reference for other materials with poor cycling performance.

## Introduction

With the development of electric vehicles, traditional energy storage equipment has been difficult to meet the demand of high-energy density and high-power density. Although lithium-ion battery has high energy density, its intrinsic factors limit its power density, which is hard to meet the requirement of the output of high power in practical application of electric vehicle. Supercapacitor is the energy storage device between dielectric capacitor and rechargeable battery exhibiting fast charge-discharge rate, high power density, low cost and good cycling stability, etc. [1–5]. Supercapacitors can fall into two diverse types by the energy storage mechanism, one is the electrical double-layer capacitors (EDLCs), dominated by the adsorption and desorption of the electrostatic charge in the interface and have high power density and stable cycling characteristics but possess limited energy density; the other is pseudocapacitors, also called Faraday capacitor which is dominated by the Faradaic redox reaction and have considerable energy density [6]. The electrode materials of EDLCs generally consist of carbon materials, such as activated carbon [7], activated carbon fiber [8], carbon nanotubes [9], and graphene [10]. While the electrode materials of the pseudocapacitors are often composed of transition metal oxides, transition metal hydroxides, or layer double hydroxides, such as RuO_2_ [11], NiO [12], Mn_2_O_3_ [13], MnO_2_ [14], Co_3_O_4_ [15], NiCo_2_O_4_ [16], CoNiO_2_ [17], Ni(OH)_2_ [18], Co(OH)_2_ [19], NiAl-layered double hydroxide (NiAl-LDH) [20], NiCo-LDH [21], CoAl-LDH [22], and so on. One of the big obstacles to limiting the practical use of supercapacitors is that they have a much smaller energy density than the secondary battery. Generally, in the same specific surface area, the pseudocapacitors can provide more than ten times storage capacity than the electric double-layer capacitance which have attracted great interest in increasing their energy densities of the pseudocapacitors to partially replace the current commercialized two-time battery. However, the pseudocapacitors suffer from the low conductivity of active materials with poor power characteristics and poor cyclic stability caused by volumetric expansion during the charge and discharge processes.

In recent years, the pseudocapacitors have received considerable progress, especially in their energy density [23–26]. The oxidized nickel-based layered double hydroxides (LDHs), also known as anionic clays, have drawn intensive attention as pseudocapacitive cathode materials because of their high theoretical capacitance, stable cycling performance, abundance in the earth, and environmentally friendly [27, 28]. However, one of the main disadvantages of LDHs is the poor conductivity, which limits their power density and the application in high-power devices in the future. For solving this problem, there is a growing number of research on the complex heterostructure nanomaterials of LDHs such as NiAl-LDHs on Ni foam [29], NiAl-LDHs@carbon nanoparticles [30], NiMn-LDHs/carbon nanotubes [31], NiCo_2_S_4_ Nanotube@NiMn LDHs [32], NiCoAl-LDH/NiCo-Carbonate hydroxide [33], NiAl-LDHs/graphene [34], and so on. Some of the above-mentioned studies have indeed increased their conductivity than individual material, but due to the introduction of conductive medias or other active materials, the energy densities of the overall active materials have a certain effect. Therefore, to find a material which has a good conductivity and can also provide a relatively better energy density to compound with LDHs is critical for LDH materials.

Many efforts have been done to focus on the anode materials of the pseudocapacitors to achieve high capacity and good cyclic behavior, including RuO_2_ [35], FeO_x_ [36–38], ZnO [39], VO_x_ [40–42], and so on. Among them, α-Fe_2_O_3_ is a promising anode material which has attracted great deal of research interest due to the appropriate potential window for rich redox chemistry, the high theoretical capacity, the relatively stable structure, low cost, and abundance in the earth. In recent advance, the reported capacity and the cycling stability of α-Fe_2_O_3_ is far below its theoretical capacity and a long way from commercial use, respectively, which can be contributed to the poor conductivity, the reduction of the active regions, and the volume changes of the electrodes during the redox reaction. In order to address these challenges, researchers have developed different nanostructures of α-Fe_2_O_3_ for improvement of electrochemical properties, which can be categorized as nanowires, nanotubes, nanosheets, nanosphere, and nanoflowers. But for all this, the transformation of crystal structure of the α-Fe_2_O_3_ is occurred in the redox reaction, which will lead to the volume changes and the reduction of the active regions. It still restrains the utilization of α-Fe_2_O_3_ in the anode of the supercapacitors. Therefore, it is very urgent to find an effective way to develop α-Fe_2_O_3_ as the anode electrode of supercapacitors with both high specific capacity and good cycling performance.

Based on the above consideration, we report a hierarchical CoNiO_2_@NiAl-LDHs core-shell nanosheet as the cathode electrode and the hollow sphere of α-Fe_2_O_3_ as the anode electrode of all-solid-state supercapacitor, which PVA&KOH was introduced as the solid electrolyte and separator. CoNiO_2_ is a kind of nickel-cobalt material oxides, which has been confirmed that it is a material with high energy density and good conductivity in our previous research work [17]. CoNiO_2_ was constructed as an inner framework for supporting the outer shell of NiAl-LDHs nanosheets. Two kinds of materials in the inside and outside are based on the nickel element as the main framework, making it easy to form a whole integrated architecture. The structure of CoNiO_2_@NiAl-LDHs effectively reduced the contact resistance and further increased the effective active area of NiAl-LDHs. The unique 3D hollow sphere of α-Fe_2_O_3_ is made up of nanoparticles, which enables each nanoparticle to be easily in contact with the all-solid-state electrolyte and provides sufficient active region for electrochemical reactions. The as-prepared CoNiO_2_@NiAl-LDHs cathode electrode exhibited a high specific capacitance of 1905 F g^−1^ at the current density of 1 A g^−1^. And even at the current density of 8 A g^−1^, the specific capacitance can be maintained at 1555 F g^−1^. Meanwhile, the hollow sphere of α-Fe_2_O_3_ anode electrode displayed a remarkable electrochemical performance, which the specific capacitance can achieve 802 F g^−1^ at 1 A g^−1^ and 70.3% of the capacity retention with the high current density of 16 A g^−1^. Two all-solid-state asymmetric supercapacitor (ASC) devices in series are assembled by the cathode electrode material of CoNiO_2_@NiAl-LDHs and the anode electrode material of α-Fe_2_O_3_ with the electrolyte of PVA&KOH, which exhibits a maximum energy density of 65.68 Wh kg^−1^ at the power density of 369.45 W kg^−1^. Moreover, the system shows a prominent cycling stability of 88.8% retained over 1000 cycles at 1 A g^−1^.

## Methods

### Materials

All the reagents were of analytical grade without any further purification. Nickel nitrate hexahydrate (Ni(NO_3_)_2_·6H_2_O), cobalt nitrate hexahydrate (Co(NO_3_)_2_·6H_2_O), aluminum nitrate nonahydrate (Al(NO_3_)_3_·9H_2_O), Iron(III) chloride hexahydrate (FeCl_3_·6H_2_O), anhydrous ethanol, and hexadecyl trimethyl ammonium bromide (CTAB) were obtained from Sinopharm Chemical Reagent Co. Sodium-p-styrenesulfonate (PSS) was gained from Aladdin Chemical Reagent Co.

### Preparation of the PVA&KOH

In a typical preparation of PVA&KOH, 6 g polyvinyl alcohol (PVA) was added to the 40 ml deionized water and was heated to 95 °C for 2 h with magnetic stirring. And then 4 g KOH was dissolved in 10 ml deionized water. The obtained KOH solution was added to the PVA solution dropwise. After that, continued stirring for 30 min and cooled down to the temperature of 30 °C. Finally, the PVA&KOH was formed.

### Synthesis of CoNiO2

In a typical synthesis procedure, firstly, the Ni(NO_3_)_2_·6H_2_O and Co(NO_3_)_2_·6H_2_O solution of 1 mol L^−1^ were prepared, respectively. And then 4 ml of Ni(NO_3_)_2_·6H_2_O and 4 ml Co(NO_3_)_2_·6H_2_O were added into 32 ml deionized water with magnetic stirring for 10 min, followed by 0.12 g PSS was dissolved in the solution with magnetic stirring for 15 min. In addition, the 36 mmol of urea was poured into the above solution with continuous magnetic stirring for 15 min. The resulting transparent solution was poured into a 50 ml Teflon kettle with stainless steel autoclave for hydrothermal reaction at 90 °C for 12 h. After the reactor was cooled down to room temperature, the products were collected and washed by centrifugation and rinsed with deionized water and ethanol for several times, respectively. The obtained products of the CoNiO_2_ precursors were dried at 60 °C for 12 h and calcinated at 300 °C in air atmosphere for 2 h at a heating rate of 5 °C min^−1^.

### Synthesis of CoNiO_2_@NiAl-LDHs

The NiAl-LDHs were grown on the prepared CoNiO_2_ to form the CoNiO_2_@NiAl-LDHs materials also by a hydrothermal method. Then, 0.2 g as-prepared CoNiO_2_ and 1.2 g hexadecyl trimethyl ammonium bromide (CTAB) was dispersed in the solutions of 20 ml deionized and 20 ml ethanol with ultrasonic dissolution for 30 min and magnetic stirring for 20 min. Then, 3 mmol Ni(NO_3_)_2_·6H_2_O and 1 mmol Al(NO_3_)_3_·9H_2_O were added; the mixture kept under magnetic stirring for 10 min, followed by 16 mmol urea was added with magnetic stirring for 15 min. And then the obtained solution was transferred to a 50 ml Teflon kettle with stainless steel autoclave for hydrothermal reaction at 90 °C for 12 h. When the reactor cooled down to room temperature, the products were collected and washed by centrifugation with deionized water and ethanol for several times. And then the obtained products were dried at 60 °C for 12 h in air atmosphere.

### Synthesis of α-Fe_2_O_3_

In a typical procedure, 10 mmol FeCl_3_·6H_2_O was dissolved into 40 ml of anhydrous ethanol and 40 ml deionized water with magnetic stirring for 30 min. The obtained transparent solution was transferred to a 100 ml Teflon kettle with stainless steel autoclave for hydrothermal reaction at 200 °C for 12 h. The products were collected and washed by centrifugation and rinsed with deionized water and ethanol for several times, respectively. And then the as-prepared products were also dried at 60 °C for 12 h in air atmosphere.

### Characterization

The as-prepared materials were characterized by X-ray diffractometer (PANalytical X’Pert Pro XRD, CuKa λ = 0.15405 nm) with a scan rate of 5° min^−1^. Morphology of the prepared products were examined by field emission scanning electron microscopy (FESEM, Inspect F, FEI) with accelerating voltage of 20 kV. Moreover, the transmission electron microscopy (TEM), the high-resolution transmission electron microscopy (HRTEM), and energy dispersive X-ray (EDX) spectroscopy data were acquired using an FEI Tecnai F20 electron microscope operated at accelerating voltage of 200 kV.

### Electrochemical Measurements

Electrochemical tests were characterized in a three-electrode electrochemical system with an aqueous electrolyte solution of 6 M KOH. A platinum foil was served as the counter electrode, and the Hg/HgO electrode was served as a reference electrode. Ni foam (110 PPI, thickness: 1.5 mm, surface density: 380 g m^−2^, Lizhiyuan Co., Ltd.) was cut to a size of 1 × 6 cm, washed with ethanol and deionized water in an ultrasonic bath, and used as a current collector. To prepare the working electrodes, sample powders, super-p, and polytetrafluoroethylene (PTFE) were combined in the mass ratio of 80:15:5 in a small amount of ethanol and formed into a homogeneous paste. The paste was then painted on Ni foam (1 cm × 1 cm) and pressed into an anode electrode sheet using hydraulic pressing at a temperature of 150 °C after drying at 80 °C in air. The mass loading of the cathode electrode and the anode electrode were about 20 mg cm^−2^, respectively. Electrochemical tests were carried out by an RST5200F electrochemical workstation (Suzhou Risetest Electronic Co., Ltd., Suzhou, China). The values of specific capacitance for the working electrodes were calculated by the following formula (1):

1 $$ C=I\Delta  t/m\Delta  V $$

The symbols of *C*, *I*, Δ*t*, *m*, Δ*V* represent the specific capacitance (F g^−1^) of the active material of the electrode, discharge current (A), discharge time (s), the mass loading of the active material (g), the potential window of discharge (V), respectively. In addition, the electrochemical impedance spectroscopy (EIS) was carried out with the AC voltage of 5 mV amplitude in the frequency range of 0.01 Hz–10 kHz at the open circuit potential.

## Results and Discussion

**Fig. 1:**
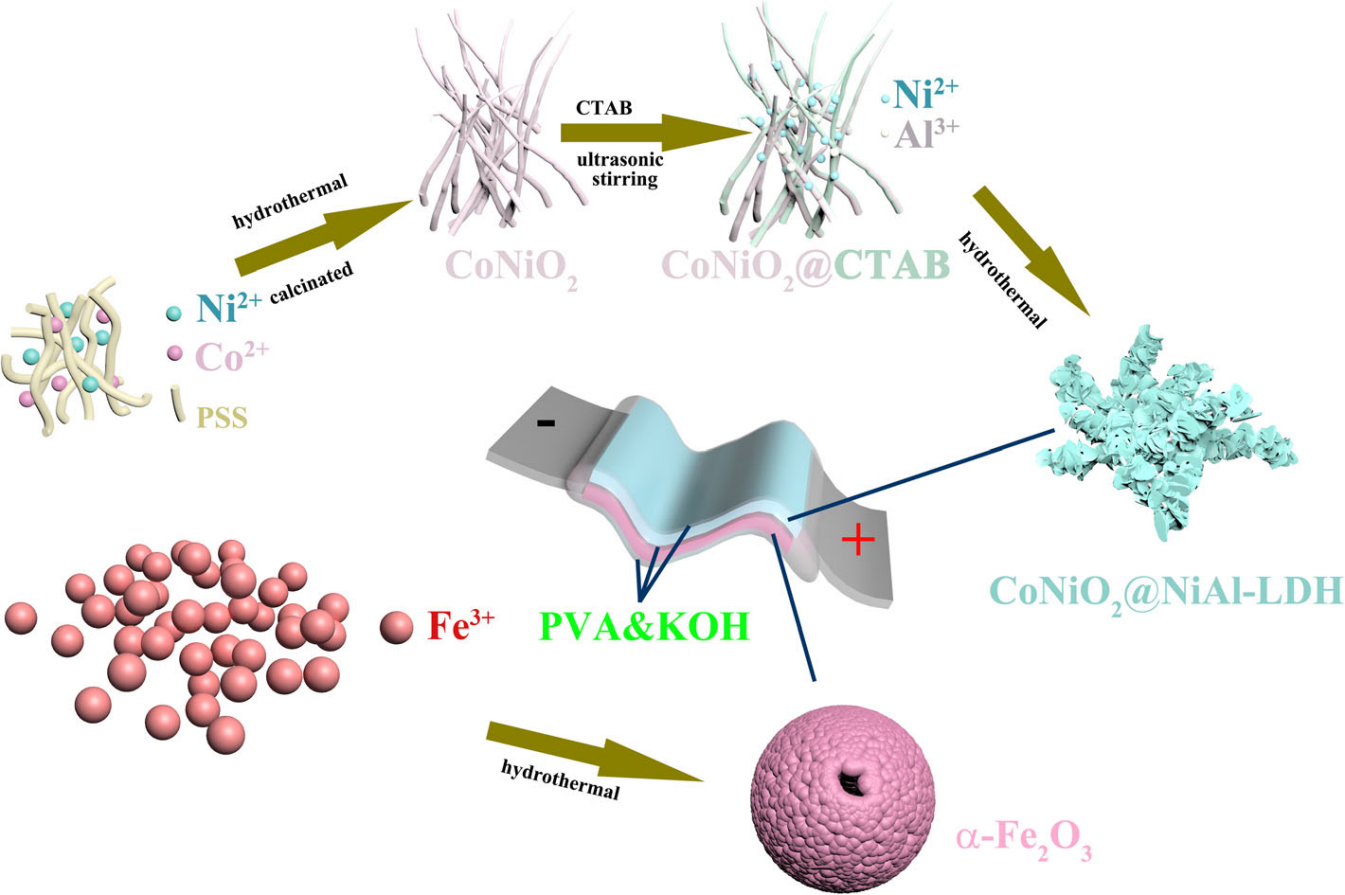
Schematic illustration of the synthesis procedure for CoNiO_2_@NiAl-LDH, the hollow sphere of α-Fe_2_O_3_, and the formed solid ASC device with the electrolyte of PVA&KOH

The schematic fabrication process is displayed in Fig. 1. Firstly, CoNiO_2_ was prepared using a hydrothermal method followed by a calcining process. And then CTAB and the CoNiO_2_ were mixed together in deionized water by stirring and ultrasonic. Subsequently, the CoNiO_2_@NiAl-LDH was obtained by another hydrothermal reaction. Meanwhile, the hollow sphere of α-Fe_2_O_3_ was grown by a hydrothermal way. The all-solid-state ASC device was fabricated by the CoNiO2@NiAl-LDH as the cathode material, the α-Fe_2_O_3_ as the anode material, the foamed nickel as the current collect collector, and the PVA&KOH as the electrolyte and the separator.

**Fig. 2:**
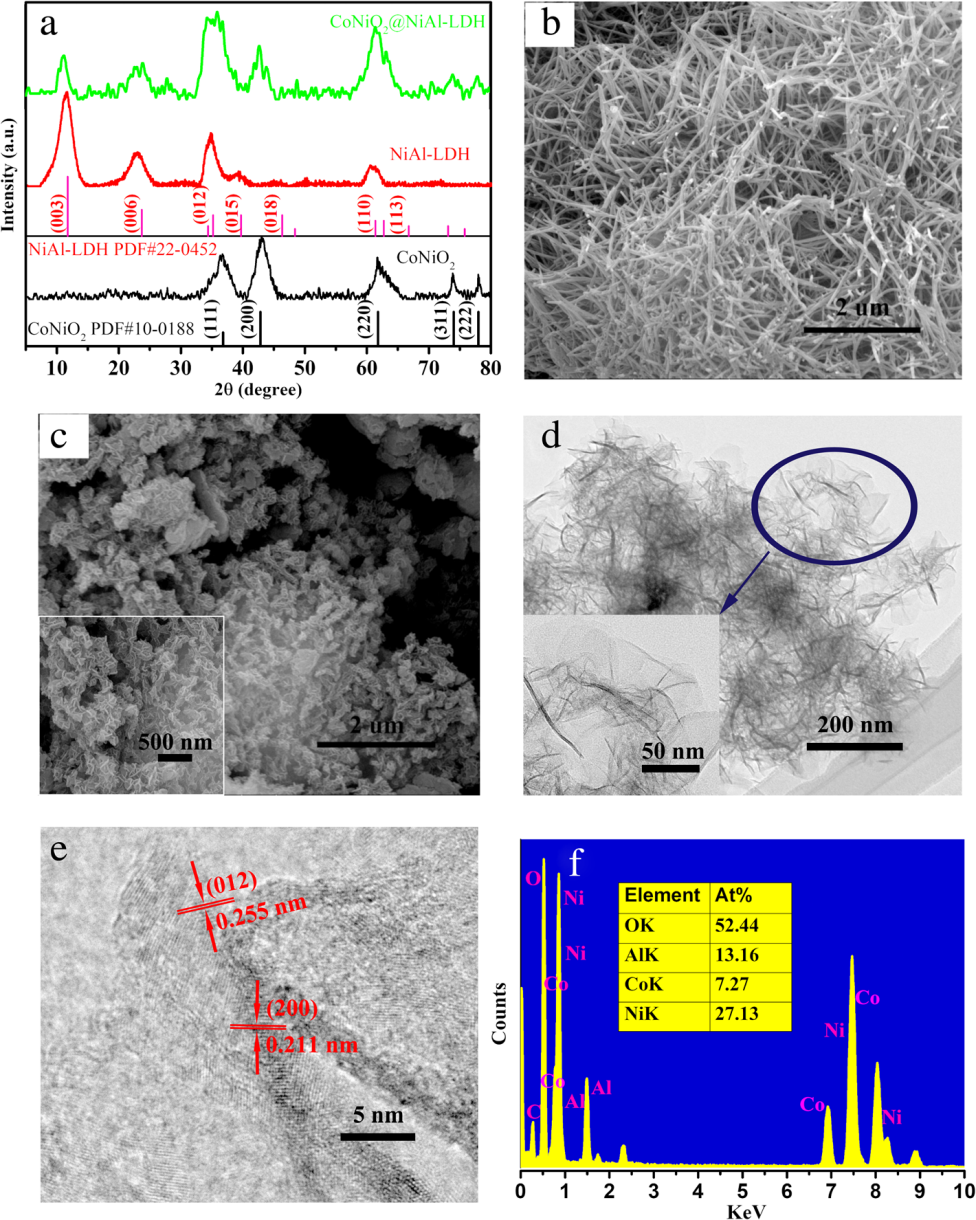
**a** XRD patterns of samples: CoNiO_2_, NiAl-LDH, CoNiO_2_@NiAl-LDH. **b**, **c** SEM image of CoNiO_2_, CoNiO_2_@NiAl-LDH. **d** TEM images of CoNiO_2_@NiAl-LDH. **e** TEM images at high magnifications. **f** The EDX spectrum of the CoNiO_2_@NiAl-LDH

The phase and composition of the samples are illustrated by X-ray diffraction (XRD) presented in Fig. 2a. The sharp peaks for the resultant CoNiO_2_ locate at 36.8°, 42.8°, 61.8°, 74.0°, and 80.0° are indexed to CoNiO_2_ (JCPDS Card no. 10-0188). And all the diffraction peaks of the resultant NiAl-LDH are in good agreement with the phase of Ni_5_Al_4_O_11_·18H_2_O (JCDPS Card no. 22-0452). After the hydrothermal growth of the NiAl-LDH, the XRD pattern of the hybrid structure (green line) contains the peaks of CoNiO_2_ and NiAl-LDH, revealing that the two phases of the obtained NiAl-LDH are coexistent on the surface of CoNiO_2_. In the hybrid structure, the intensity of the diffraction peaks relative to the CoNiO_2_ decreases, which is likely that the NiAl-LDH is distributed on the surface of CoNiO_2_ nanostructure and concealed by the strong diffraction peaks of the NiAl-LDH. Figure 2b, c presents the SEM images of the as-synthesized CoNiO_2_ and CoNiO_2_@NiAl-LDH, which reveal that the CoNiO_2_ with a diameter of approximately 40 nm were interlaced with each other to construct a three-dimensional structure and facilitate the transport of electrons inside of active materials. Compared with the CoNiO_2_, the NiAl-LDH nanosheets were grown on the surface of the CoNiO_2_ to form the CoNiO_2_@NiAl-LDH with a diameter of approximately 100 nm as shown in Fig. 2c which the interconnected nanosheets may promote the efficient contact of electrolyte ions. It can be seen from the TEM image in Fig. 2d that the diameter of CoNiO_2_@NiAl-LDH is mainly consistent with that obtained by the SEM images. Meanwhile, in Fig. 2d, the nanosheets can be clearly observed; however, the framework of CoNiO_2_ is hard to find, which probably the CoNiO_2_ recrystallized and combined with NiAl-LDH during the hydrothermal reaction of growing NiAl-LDH. The interplanar spacings is displayed in the high-resolution TEM (HRTEM) images in Fig. 2e, which the measured values are 0.211 nm and 0.255 nm, corresponding to (200) planes of CoNiO_2_ and (012) planes of NiAl-LDH, respectively. Figure 2f shows the energy dispersive X-ray (EDX) that indicates the existence of O, Al, Co, and Ni elements, which the atomic ratio is corresponding well with the atomic ratio of the CoNiO_2_@NiAl-LDH.

**Fig. 3:**
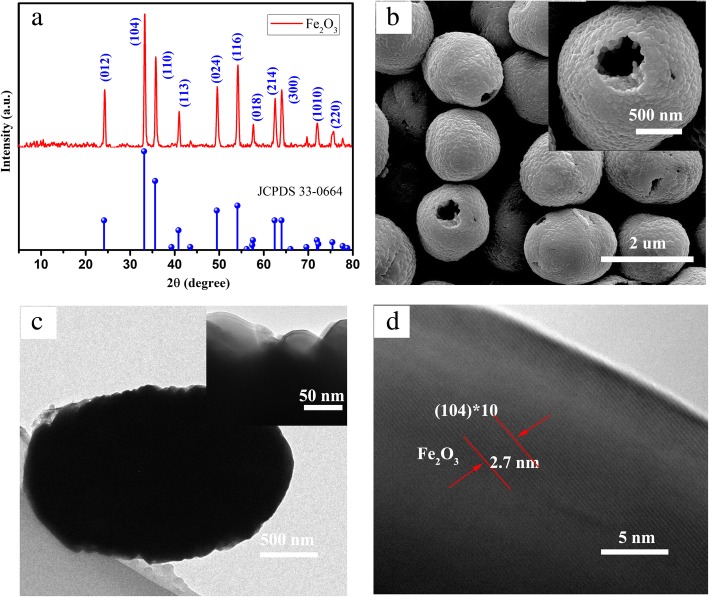
**a** XRD pattern of samples: α-Fe2O3. **b** SEM image of α-Fe_2_O_3_. **c** TEM images of α-Fe_2_O_3_. **d** TEM images at high resolution

The as-synthesized hollow sphere α-Fe_2_O_3_ is characterized by powder XRD analysis, as observed in Fig. 3a. The distinct diffraction peaks are detected at 2θ values of 24.1°, 33.2°, 35.6°, 40.8°, 49.5°, 54.1°, 57.6°, 62.4°, 64.0°, 71.9°, and 75.4°, which can be indexed to (012), (104), (110), (113), (024), (116), (018), (214), (300), (1010), and (220) plane reflections of the hematite (α-Fe_2_O_3_ JCPDS Card no. 33-0664). No other peaks are detected from possible impurities, such as the diffraction peaks of Fe_3_O_4_, FeOOH, or γ-Fe_2_O_3_, which indicates that the as-prepared hollow sphere α-Fe_2_O_3_ has a high purity. Morphological features of as-prepared α-Fe_2_O_3_ are studied by SEM and TEM as illustrated in Fig. 3b–d. It is evident that the diameter of the hollow sphere α-Fe_2_O_3_ is approximately 1.5 μm from Fig. 3b. Further observation finds that the hollow sphere α-Fe_2_O_3_ is made up of nanoparticles with an average size of 70–80 nm which is also clearly observed from the edge of the as-prepared α-Fe_2_O_3_ in the TEM images of Fig. 3c. The high resolution of the TEM images shows the interplanar spacing of the hollow sphere α-Fe_2_O_3_, as observed in Fig. 3d, which the measured value is 0.27 nm, corresponding to (104) plane of α-Fe_2_O_3_.

**Fig. 4:**
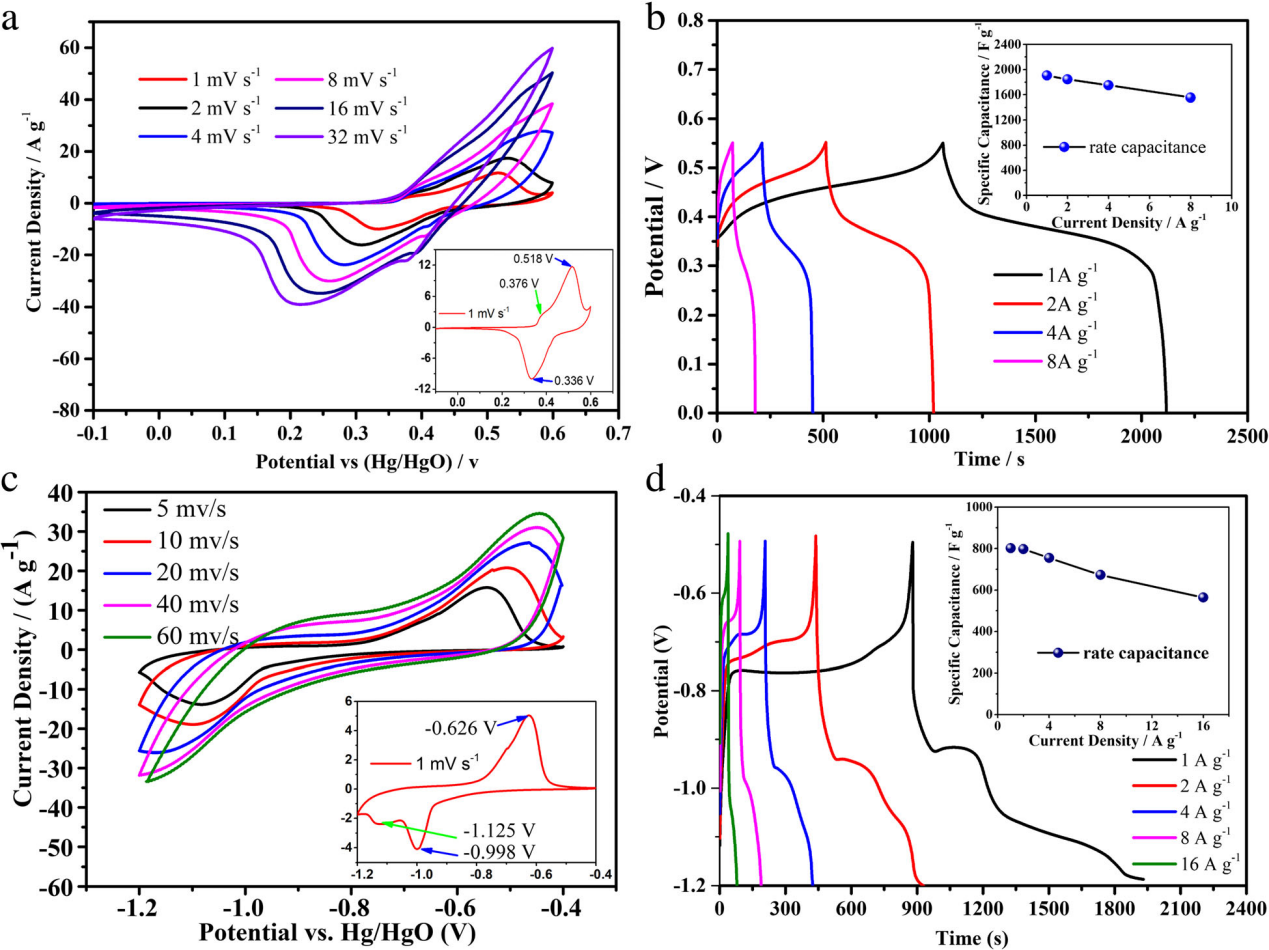
**a** Cyclic voltammograms of CoNiO_2_@NiAl-LDH composites. **b** Galvanostatic charge-discharge curves of CoNiO_2_@NiAl-LDH electrode at different current densities (1, 2, 4, 8 A g^−1^). **c** Cyclic voltammograms of the hollow sphere α-Fe_2_O_3_. **d** Galvanostatic discharge-charge curves of the hollow sphere α-Fe_2_O_3_ electrode at different current densities (1, 2, 4, 8, 16 A g^−1^)

To evaluate the electrochemical characteristics of CoNiO_2_@NiAl-LDH and α-Fe_2_O_3_ as electrode materials, the electrochemical tests are performed in a three-electrode system in 6 M KOH aqueous electrolyte, which the Hg/HgO and Pt are employed as the reference electrode and the counter electrode, respectively. Figure 4a shows the CV curves of CoNiO_2_@NiAl-LDH electrode at the scan rate of 1, 2, 4, 8, 16, and 32 mV s^−1^ in the potential window of − 0.1 to 0.6 V (vs. Hg/HgO). It can be found that the CV curves at different scan rate basically share a similar shape, whereas with the increase of the scan rate, the oxidation peaks slowly shift toward the positive voltage and the reduction peaks slowly shift toward the negative voltage, attributed to the polarization effect of the electrode [5]. The peak are merely shifted by 0.11 V when the scan rate is increased from 1 to 32 mV s^−1^, indicating a low resistance of the CoNiO_2_@NiAl-LDH electrode. Meanwhile, with the increase of the scan rate, the peak current increases, implying the rapid electron and ion transfer rates. The CV curve is exhibited at the scan rate of 1 mV s^−1^ in the bottom-right side of Fig. 4a. It is evident that some of redox peaks were observed, such as the oxidation peak at 0.518 V and the reduction peak at 0.336 V, which is primarily due to the redox reaction of the Ni [43]. Meanwhile, the oxidation peak at 0.378 V was also detected in the CV measurements, implying that the Co specie also plays a role in the redox reaction at the scan rate of 1 mV s^−1^. However, with the increase of the scan rate, the oxidation peak of Co is disappeared, which indicates that the Co specie may exist mainly as a conducting medium in the nanocomposites of CoNiO_2_@NiAl-LDH.

The galvanostatic charge-discharge measurements of CoNiO_2_@NiAl-LDH electrode is conducted at the current densities of 1 A g^−1^, 2 A g^−1^, 4 A g^−1^, and 8 A g^−1^ with a potential window between 0 and 0.55 V (vs Hg/HgO), as presented in Fig. 4b. There is a distinct platform region during the charge and discharge, which suggests the faradaic pseudocapacitive behavior of the electrodes. At the same current densities, the specific capacitances are calculated by the formula (1), as shown in the top-right side of Fig. 4b, which exhibits a high specific capacitance of 1905 F g^−1^ at the current density of 1 A g^−1^ and much larger than our former research of CoNiO_2_ (1462 F g^−1^) [17] and NiAl-LDH (1313 F g^−1^) [44]. The specific capacitance retention is up to 81.6% at the current density of 8 A g^−1^, implying the electrode of CoNiO_2_@NiAl-LDH has an excellent rate capability and it can be attributed to the core-shell nanostructure. The outstanding electrochemical performance of CoNiO_2_@NiAl-LDH is better than most of previously reported for bimetallic oxides and the layer double hydroxides, especially the high discharge voltage plateau, such as NiCo_2_O_4_@MnMoO_4_ [45], NiP@CoNi-LDH [46], NiCo-LDH/NiCoP@NiMn-LDH [47], MnCo_2_O_4_@Co_3_O_4_ [48], and so on.

The CV curves of the as-prepared α-Fe_2_O_3_ are measured at the scan rate of 5, 10, 20, 40, and 60 mV s^−1^ in the potential window of − 1.2 to − 0.4 V (vs. Hg/HgO) in Fig. 4c. It can be seen that the CV curves also share a similar shape with a pair of the oxidation peak and the reduction peak, indicating the behavior of the pseudocapacitance. The oxidation peak are only just shifted by 0.1 V when the scan rate is increased from 5 to 60 mV s^−1^, implying a very low resistance of the as-prepared α-Fe_2_O_3_ electrode. The CV curve with a slow scan rate of 1 mV s^−1^ is displayed in the bottom-right side of Fig. 4c. It can be observed that the oxidation peak is − 0.626 V and the reduction peak is − 0.998 V, corresponding to the conversion between Fe^3+^ and Fe^2+^ [49]. But at the same time, the reduction peak of − 1.125 V is also observed, which probably some of the materials involved in the conversion between Fe^3+^ and Fe [50].

The galvanostatic discharge-charge tests of α-Fe_2_O_3_ electrode is carried out at the current densities of 1 A g^−1^, 2 A g^−1^, 4 A g^−1^, 8 A g^−1^, and 16 A g^−1^ with a potential window between − 0.1 and − 1.2 V (vs Hg/HgO), as presented in Fig. 4d. It can be also found that a distinct platform region is occurred during the charge and discharge, indicating the faradaic pseudocapacitive behavior of the as-prepared α-Fe_2_O_3_ electrodes. The specific capacitances are calculated at the current density of 1, 2, 4, 8, and 16 A g^−1^, as shown in the top-right side of Fig. 4d. The specific capacitance of 1 A g^−1^ is 802 F g^−1^ and only 29.7% loss of the capacitance is acquired at the current density of 16 A g^−1^, where the rate capability is higher than that of Fe_2_O_3_ based on previous work [51–55].

**Fig. 5:**
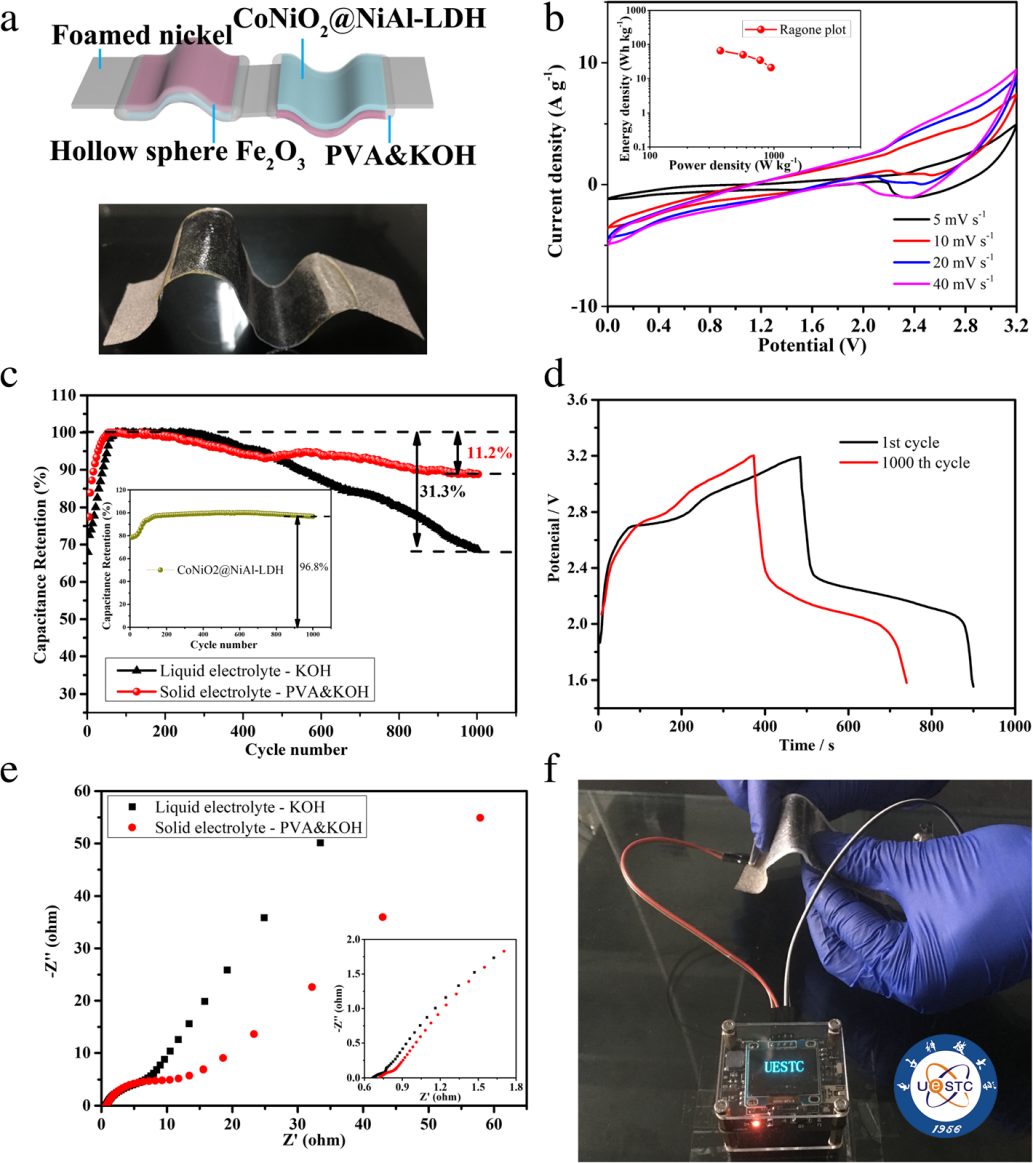
**a** The assembly schematic of two solid ASC devices in series and photograph of the as-prepared two solid ASC devices in series. **b** Cyclic voltammograms of two solid CoNiO_2_@NiAl-LDH//α-Fe_2_O_3_ in series and the ragone plots in the top-left side. **c** Cycling performance of the liquid ASC devices and the solid ASC devices of CoNiO_2_@NiAl-LDH//α-Fe_2_O_3_ at the current density of 1 A g^−1^. **d** The comparison of the galvanostatic charge-discharge measurements between the 1st and 1000th in 1 A g^−1^. **e** The comparison of the Nyquist plots of the liquid ASC devices and the solid ASC devices. **f** Photograph of an OLED display powered by the two solid ASC in series

The assembly schematic two solid ASC devices in series (named as TSASC) is shown in Fig. 5a. It can be seen that the foamed nickel is regarded as the current collector, the hollow sphere as the material of the anode electrode, the CoNiO_2_@NiAl-LDH as the material of the cathode electrode, and the PVA&KOH as the separator and the solid electrolyte. In addition, only one foamed nickel (a cathode electrode at one end and an anode electrode at the other end of the nickel foam) is used as the connection part of TSASC, which can reduce the contact resistance at the junction of two electrodes. The photograph of the as-prepared TSASC is displayed in the bottom of Fig. 5a, implying a good flexibility of the devices.

The cyclic voltammograms of two solid CoNiO_2_@NiAl-LDH//α-Fe_2_O_3_ in series are conducted at the scan rate of 5 mV s^−1^, 10 mV s^−1^, 20 mV s^−1^, and 40 mV s^−1^ with the potential window between 0 and 3.2 V, as shown in Fig. 5b. It can be observed that the CV curves share a similar shape and the position of redox peak is nearly no shift with the scan rates increased, suggesting a good rate capability of the ASC devices. And the Ragone plots of the two solid CoNiO_2_@NiAl-LDH//α-Fe_2_O_3_ in series ASC devices are shown in the bottom of Fig. 4b which the power density and the energy density are calculated by the CV curves at the scan rate of 5 mV s^−1^, 10 mV s^−1^, 20 mV s^−1^, and 40 mV s^−1^. The CoNiO_2_@NiAl-LDH//α-Fe_2_O_3_ TSASC devices exhibit a maximum energy density of 65.7 Wh kg^−1^ with the power density of 369.45 W kg^−1^ at the CV scan rate of 5 mV s^−1^ and even at the scan rate of 10 mV s^−1^, the energy density can still be remained 21.1 Wh kg^−1^ with the power density of 946.35 W kg^−1^ based on the sum of the total mass of cathode and anode active substances, indicating a considerable rate capability in the two all-solid state in series devices.

The electrochemical stabilities of the CoNiO_2_@NiAl-LDH//α-Fe_2_O_3_ TSASC devices and the two liquid CoNiO_2_@NiAl-LDH//α-Fe_2_O_3_ in series ASC (named as TLASC) devices are evaluated by the galvanostatic charge-discharge measurements with the current density of 1 A g^−1^ for 1000 cycles, as shown in Fig. 5c. The CoNiO_2_@NiAl-LDH//α-Fe_2_O_3_ TSASC devices exhibit remarkable cycling stability with 88.8% specific capacitance retention while the CoNiO_2_@NiAl-LDH//α-Fe_2_O_3_ TLASC devices only achieve 68.7% after 1000 cycles. Additionally, the cycling stability of CoNiO_2_@NiAl-LDH for the cathode materials is presented in the embedded chart of Fig. 5c, indicating an excellent cycling stability with 96.8% retention of the specific capacitance. That means the attenuation of cyclic performance for the ACS devices are mainly caused by the instability of the anode material of α-Fe_2_O_3_ in a continuous redox reaction [56]. Compared the cycling stability of TSASC and TLASC, it can be found that the solid electrolyte of PVA&KOH can increase the stability of the anode material of α-Fe_2_O_3_, which can be probably that the solid electrolyte of PVA&KOH limits the expansion and passivation of α-Fe_2_O_3_ during the continuous redox reaction. It is equivalent to solving the problem of iron oxide cycling performance in an alternative way, and of course it can be analogue to other materials with poor cycling performance.

The comparison of galvanostatic charge-discharge tests with the current density of 1 A g^−1^ between the origin cycle and 1000th cycle is presented in Fig. 4d. It can be seen that both of two galvanostatic charge-discharge curves have charging and discharging platforms, suggesting a Faradaic reaction. The discharge platform of the 1st cycle is higher than that of the 1000th cycle and the discharge time of the 1st cycle is longer than that of 1000th cycle, which indicates that the electrode materials still have a certain degree of passivation, although the PVA&KOH can prevent some passivation of the electrode materials. Meanwhile, the charge platform of the 1000th cycle is higher than that of the 1st cycle, implying the increase of the internal resistance of the ASC devices.

Further studying the electrochemical properties of the CoNiO_2_@NiAl-LDH//α-Fe_2_O_3_ TLASC and TSASC devices, the electrochemical impedance (EIS) is carried out at the open circuit potential. Figure 5e shows the Nyquist plots of the CoNiO_2_@NiAl-LDH//α-Fe_2_O_3_ TLASC and TSASC devices in the frequency range of 0.01 Hz to 100 kHz with an AC perturbation of 5 mV. The Nyquist plots can be divided into two distinct parts of a semicircle in the high-frequency region and a straight line in the low-frequency region. And the intercepts of the Nyquist plots on the real axis are about 0.67 and 0.72 Ω for the CoNiO_2_@NiAl-LDH//α-Fe_2_O_3_ TLASC and TSASC, respectively, indicating the extremely low intrinsic resistances. The intrinsic resistances are lower than most of previous reported Fe_2_O_3_-based ASC devices [57–60]. Comparing to the Nyquist plots of CoNiO_2_@NiAl-LDH//α-Fe_2_O_3_ TLASC and TSASC, it can be found that the small semicircle diameter of the TSASC is larger than that of TLASC, suggesting that the solid electrolyte of PVA&KOH has a certain barrier for electron transportation. It is proved that the conductivity of one-dimensional nanostructures is better than that of bulk materials [37]. The interlaced nanowires of CoNiO_2_ can act as the channel for efficient electron transportation between the NiAl-LDH and the current collectors and the hollow sphere structure of α-Fe_2_O_3_, composed of nanoparticles, also possesses good conductivity and the ability to efficient transport electron. It is the reason that the Nyquist plots shows low intrinsic resistances and good charge-transfer abilities. An organic light-emitting diode (OLED) display with drive circuit is used to simulate the application of the CoNiO_2_@NiAl-LDH//α-Fe_2_O_3_ TSASC on the mobile phone, which the TSASC successfully powered the OLED display and the brightness of the screen is very high as shown in Fig. 5e.

## Conclusion

In summary, a template-free hierarchical nanostructure CoNiO_2_@NiAl-LDH nanocomposite and α-Fe_2_O_3_ with a hollow spherical structure composed of nanoparticles were successfully prepared. The cathode electrode of CoNiO_2_@NiAl-LDH exhibits outstanding electrochemical performance with a high specific capacitance of 1905 F g^−1^ at 1 A g^−1^ and also possesses 1555 F g^−1^ (81.6% of initial capacitance) at the high current density of 8 A g^−1^, implying a good rate capability, which can be ascribed to efficient electron transportation channel and the high utilization rate of the active materials. The anode electrode of hollow sphere α-Fe_2_O_3_ reveals excellent electrochemical properties with a specific capacitance of 802 F g^−1^ at 1 A g^−1^ and the capacitance retention of 70.3% at the high current density of 16 A g^−1^ which also shows a good rate capability due to the good conductivity and the ability of the hollow spherical structure composed of nanoparticles to efficient transport electron. The PVA&KOH was utilized as the separator and the solid electrolyte of the two all-solid-state ACS devices in series which has been constructed by the cathode electrode of CoNiO_2_@NiAl-LDH and the anode electrode of hollow sphere α-Fe_2_O_3_. The CoNiO_2_@NiAl-LDH//α-Fe_2_O_3_ TSASC exhibits a maximum energy density of 65.68 Wh kg^−1^ at the power density of 369.45 W kg^−1^. Moreover, the system shows an enhanced cycling stability of 88.8% retained over 1000 cycles at the current density of 1 A g^−1^ which is compared to previously reported ASC devices. Moreover, in addition to many advantages that the solid electrolyte in ASC devices already have, it can be interestingly found that it can be an alternative way of solving the problem of iron oxide cycling performance, and of course it can be analogue to other materials with poor cycling performance.

## Abbreviations

ASCs: Asymmetric supercapacitors
CTAB: Hexadecyl trimethyl ammonium bromide
EDLCs: Electrical double-layer capacitors
EDX: Energy dispersive X-ray
EIS: Electrochemical impedance spectroscopy
FESEM: Field emission scanning electron microscopy
HRTEM: High-resolution transmission electron microscopy
NiAl-LDH: NiAl-layered double hydroxide
OLED: Organic light-emitting diode
PSS: Sodium-p-styrenesulfonate
PTFE: Polytetrafluoroethylene
PVA: Polyvinyl alcohol
TEM: Transmission electron microscopy
TLASC: Two liquid ASC devices in series
TSASC: Two solid ASC devices in series
XRD: X-ray diffractometer
